# The Molecular Evolution of the p120-Catenin Subfamily and Its Functional Associations

**DOI:** 10.1371/journal.pone.0015747

**Published:** 2010-12-31

**Authors:** Robert H. Carnahan, Antonis Rokas, Eric A. Gaucher, Albert B. Reynolds

**Affiliations:** 1 Department of Cancer Biology, Vanderbilt University, Nashville, Tennessee, United States of America; 2 Department of Biological Sciences, Vanderbilt University, Nashville, Tennessee, United States of America; 3 School of Biology, Georgia Institute of Technology, Atlanta, Georgia, United States of America; University of Colorado, Boulder, United States of America

## Abstract

**Background:**

p120-catenin (p120) is the prototypical member of a subclass of armadillo-related proteins that includes δ-catenin/NPRAP, ARVCF, p0071, and the more distantly related plakophilins 1–3. In vertebrates, p120 is essential in regulating surface expression and stability of all classical cadherins, and directly interacts with Kaiso, a BTB/ZF family transcription factor.

**Methodology/Principal Findings:**

To clarify functional relationships between these proteins and how they relate to the classical cadherins, we have examined the proteomes of 14 diverse vertebrate and metazoan species. The data reveal a single ancient δ-catenin-like p120 family member present in the earliest metazoans and conserved throughout metazoan evolution. This single p120 family protein is present in all protostomes, and in certain early-branching chordate lineages. Phylogenetic analyses suggest that gene duplication and functional diversification into “p120-like” and “δ-catenin-like” proteins occurred in the urochordate-vertebrate ancestor. Additional gene duplications during early vertebrate evolution gave rise to the seven vertebrate p120 family members. Kaiso family members (i.e., Kaiso, ZBTB38 and ZBTB4) are found only in vertebrates, their origin following that of the p120-like gene lineage and coinciding with the evolution of vertebrate-specific mechanisms of epigenetic gene regulation by CpG island methylation.

**Conclusions/Significance:**

The p120 protein family evolved from a common δ-catenin-like ancestor present in all metazoans. Through several rounds of gene duplication and diversification, however, p120 evolved in vertebrates into an essential, ubiquitously expressed protein, whereas loss of the more selectively expressed δ-catenin, p0071 and ARVCF are tolerated in most species. Together with phylogenetic studies of the vertebrate cadherins, our data suggest that the p120-like and δ-catenin-like genes co-evolved separately with non-neural (E- and P-cadherin) and neural (N- and R-cadherin) cadherin lineages, respectively. The expansion of p120 relative to δ-catenin during vertebrate evolution may reflect the pivotal and largely disproportionate role of the non-neural cadherins with respect to evolution of the wide range of somatic morphology present in vertebrates today.

## Introduction

The integration over time of increasingly sophisticated signaling and cell-cell adhesion mechanisms has likely been an essential and ongoing process in the evolution of complex metazoan life. Interestingly, the Wnt signaling and cadherin-based adhesion functions of β-catenin have coexisted at least as far back as the origin of animals [1] (though *C. elegans* is a notable exception [2]), with coordination of these roles by a single protein perhaps facilitating evolution of the first multicellular organisms. Indeed, the evolutionary importance of β-catenin is reflected by phylogenetic analyses, which suggest a widespread and persistent stabilizing selection on each of the Armadillo (Arm) repeat sequences from Cnidarian to mouse β-catenin [3], and virtually no change in β-catenin over the ∼400 million year course of vertebrate evolution [3]. In vertebrates, β-catenin (or Plakoglobin) coexists with two other so-called “catenins” (i.e., p120-catenin and α-catenin) that together form a regulatory protein complex on the cytoplasmic tail of classical cadherins (i.e., Type I and type II cadherins). Evolutionary histories for cadherin- and β-catenin-families have been studied extensively [3], [4], [5], [6], [7], [8] but similar analyses for the p120-catenin (hereafter p120) and α-catenin families have yet to be reported.

The appearance of cadherins is clearly a watershed event in metazoan evolution. While adhesion *per se* likely predates metazoans [6], the origin and diversification of the greater cadherin family has permitted an explosion in functional diversity of intercellular interactions. Interestingly, vertebrate evolution has favored a particular paradigm, the classical cadherin, which has duplicated and reduplicated from a single vertebrate ancestor [5] to form a 26-member family. Structurally, the “classical cadherin” is comprised of five extracellular cadherin (EC) repeats and a highly conserved cytoplasmic tail containing a p120-binding juxtamembrane domain (JMD) and a C-terminal “catenin binding domain” (CBD) that interacts with β-catenin. As the predominant cadherin type in vertebrate cell-cell adhesion, the classical cadherins have also taken on fundamentally important roles in cell-cell adhesion, development and cancer, and mediate the majority of cell- and tissue-specific interactions in vertebrates.

In vertebrates, p120 behaves as a master regulator of classical cadherin stability, and is critical for proper cell-cell adhesion in most solid tissues [9], [10], [11]. Deletion (or knockdown) of the p120 gene in vertebrates (e.g., mouse, xenopus, zebrafish) is embryonic lethal despite the presence of ARVCF, δ-catenin, and p0071, closely related family members with at least partially overlapping functions [12], [13], [14], [15]. Paradoxically, the single p120 family member in invertebrates (e.g., *Drosophila melanogaster*, *Caenorhabditis elegans*) is not essential for life in most species (although this point has been debated in drosophila) [16], [17], [18]. Thus, in vertebrates, p120 has evolved one or more essential functions relative to its invertebrate counterpart, and a critical role with respect to the classical cadherins.

**Figure 1:**
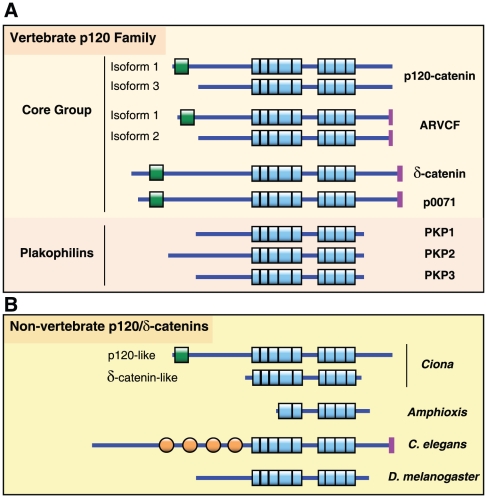
Domain structure of the p120-catenin family. (A) The vertebrate members of the p120-catenin family of proteins all contain a central Armadillo repeat domain consisting of 9 tandemly linked imperfect 42 amino-acid repeats (blue boxes). The four “Core” members also contain an amino-terminally located coiled-coil domain (green boxes). In the case of p120 and ARVCF, alternative splicing in this region gives rise to two major isoforms, which either contain (isoform 1) or not (isoform 3) this coiled-coil region. All “Core” members, except p120, also have a carboxy-terminally located PDZ ligand domain (purple boxes). (B) The invertebrate members of the p120-catenin family similarly possess centrally located Armadillo repeats, though in the case of *Amphioxis* this region contains 6 rather than 9 repeats. N-terminal regions show more diversity with no distinct domain structure (*D. melanogaster*, *Amphioxis*, *Ciona* δ-catenin-like), Fibronectin type III domains (orange circles, *C. elegans*), or a coiled-coil domain (*Ciona* p120-like). Similar to vertebrate members, the *C. elegans* family member also contains a carboxy-terminally located PDZ ligand domain (purple box).

p120 family members share a conserved central domain composed of 9 Arm repeats and flanking N- and C-terminal regions that diverge from one another (Figure 1). The “core” family members interact in adherens junctions with classical cadherins via Arm repeats 1–6 [19]. In contrast, the more distantly related plakophilins have evolved specialized roles in desmosomal junctions, which are mechanistically and spatially distinct from the adherens junction [20]. Surprisingly, despite structural similarity to p120, their interaction with desmosomal cadherins is not mediated by the Arm domain, but occurs instead through the plakophillin N-terminal head domain [21], [22], [23]. Knockout studies in mice reveal that plakophillin 2 ablation is embryonic lethal [24] while plakophilins 1 and 3 can be eliminated with relatively little effect [20], [25].

p120 also interacts directly with the transcription factor Kaiso [26]. Kaiso belongs to a large family of BTB/ZF proteins, most of which are important in development and cancer, and a closely related Kaiso subfamily consisting of Kaiso, ZBTB38 and ZBTB4 and [27]. Interestingly, Kaiso is bimodal in that it interacts with a conventional sequence-specific DNA motif referred to as the Kaiso Binding Site (KBS) [28] and also with methyl-CpG containing motifs [29]. The latter are high affinity interactions that have been reported to suppress the transcription of several tumor suppressors (e.g., pRb, p16, HIC) through interaction with inappropriately methylated CpG islands [30]. Kaiso has also been shown in *Xenopus* to suppress several Wnt pathway genes (e.g., Wnt 11, Siamois) by association with the KBS [31], [32]. Interestingly, a third mechanism has been proposed that does not involve direct interaction with DNA. Instead, Kaiso binds TCF, a β-catenin-associated transcription factor. Kaiso and TCF associate with one another via their DNA binding motifs, thereby mutually excluding interaction with chromatin [33]. According to this scenario, p120 may interact with and/or modulate canonical Wnt signaling via regulation of Kaiso. Indeed, overexpressed p120 promotes translocation of Kaiso out of the nucleus [31], [34], potentially facilitating TCF interaction with chromatin.

A feature shared by most, if not all members of the p120 family is physical and/or functional interaction with a number of Rho-GTPases, -GEFs and -GAPs [35]. For example, p120 can inhibit RhoA directly [36], [37], or indirectly through p190RhoGAP [38], and has been shown to promote Rac1 activation [39], [40]. In general, these activities are thought to play critical roles in regulating the cytoplasmic interface between the various cadherin receptors and the cytoskeleton.

Here, we have analyzed proteomes from 14 diverse metazoan species to understand the evolution of the p120 protein family and the origin of its functional association with classical cadherins and Kaiso. We find that all invertebrates as well as several early-branching chordate lineages contain a single family member with a “δ-catenin-like” set of functions, suggesting that the p120-family ancestor was “δ-catenin-like” and highly conserved in pre-vertebrate metazoans. Gene duplications in chordate and vertebrate evolution gave rise to the six-seven family members in present day vertebrates, and provided the raw material and opportunity for functional diversification.

Together with phylogenetic studies of the classical cadherins, our data suggest that p120- and δ-catenin-like lineages split from one another in chordates and then separately co-evolved with non-neural (E- and P-cadherin) and neural (N- and R-cadherin) branches, respectively, of the vertebrate classical cadherins. A similar scenario with respect to α-catenin (also called α-catenin-1) and α-N-catenin (neural α-catenin, also called α-catenin-2) (E. Gaucher, personal communication) suggests that these distinct branches of the (vertebrate) classical cadherin family co-evolved with their own distinct subsets of both p120 (p120 vs. δ-catenin) and α-catenin (α-catenin vs. α-N-catenin) family members. Thus, the rapid expansion of p120, relative to δ-catenin, during vertebrate evolution may in large part reflect the broader spectrum of tissue and organ diversity outside of the nervous system. Other p120-specific innovations of note include the evolution of alternative splicing relevant to epithelial to mesenchymal transformation (EMT), loss of the C-terminal PDZ ligand motif, and interaction with Kaiso. The vertebrate specific appearance of Kaiso and its unique interactions with p120, TCF4 and methyl-CpG DNA suggest other p120 connections relevant to Wnt signaling and vertebrate-specific mechanisms of transcriptional regulation.

## Results

### p120 protein family

A total of 65 protein sequences were retrieved from the 14 species examined (Table 1). All protostome species examined and early-branching chordates (the cephalochordate *Branchiostoma floridae* and the echinoderm *Strongylocentrotus purpuratus*) contain a single member from the δ-catenin family. The urochordates (represented by *Ciona intestinalis*), the closest evolutionary relatives of vertebrates, contain two members, whereas all vertebrate species contain typically seven protein members of the p120-catenin family. The only exceptions within the vertebrates are *X. tropicalis*, whose proteome contains six proteins, and the two fish, *D. rerio* and *T. rubripes*, whose proteomes contain 10 and 13 members of the p120-catenin family, respectively, almost twice the number of members of the protein family as the non-fish vertebrates (Table 1).

**Table 1:** Distribution of δ-catenin protein family members across animal phyla.

Species	Phylum	Number
Human	Vertebrata	7
Mouse	Vertebrata	7
Dog	Vertebrata	7
Chicken	Vertebrata	7
Frog (*Xenopus laevis*)	Vertebrata	6
Pufferfish	Vertebrata	13
Zebrafish	Vertebrata	10 (two of the genes are identical in sequence)
Tunicate	Urochordata	2
Amphioxus	Cephalochordata	1
Echinoderm	Echinodermata	1
Fly	Arthropoda	1
Worm	Nematoda	1
Polychaete (*Capitella*)	Annelida	1
Clam (*Lottia*)	Mollusca	1

**Figure 2:**
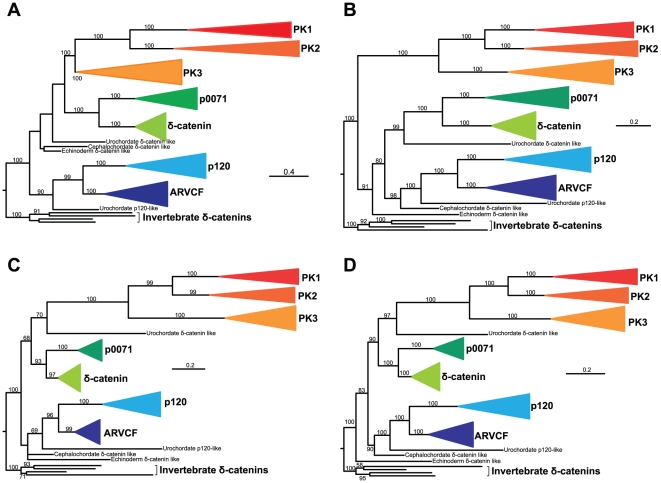
The evolutionary history of the p120-catenin family of proteins. (A) Maximum likelihood analysis on an alignment constructed using entire protein sequences, (B) Bayesian inference analysis on an alignment constructed using entire protein sequences, (C) Maximum likelihood analysis on an alignment constructed using the nine Arm domains, and (D) Bayesian inference analysis on an alignment constructed using the nine Arm domains. Color-coded clades correspond to the seven family members found in vertebrates (p120, ARVCF, p0071, δ-catenin, plakophilins 1–3) and each clade contains only sequences from vertebrates. Numbers near internodes indicate bootstrap (for maximum likelihood analyses)/posterior probability (for Bayesian inference analyses) clade support values. Clade support values <50% are not shown. PK1-3: plakophilins 1–3.

Phylogenetic analysis of all protein family members identified suggests that the seven protein members typically found in vertebrates correspond to the seven delta-catenin protein subfamilies previously identified in humans (Figures 1 and 2) [35]. Specifically, these are the plakophilin 1, plakophilin 2, plakophilin 3, p0071, delta, p120 and ARVCF subfamilies. These seven subfamilies are robustly placed into three major clades: the first clade is composed of plakophilin 1, plakophilin 2, and plakophilin 3, the second clade of δ-catenin and p0071, and the third clade of p120 and ARVCF. The phylogeny of protein members within each one of these seven functional categories is consistent with the vertebrate phylogeny, suggesting that the vertebrate ancestor possessed a single protein from each of the seven functional categories. Interestingly, the proteome of *C. intestinalis*, the closest relative of vertebrates included in this study, contains only two proteins. One protein consistently groups with the p120 – ARVCF clade (Figure 2), whereas the other protein is nested within the deuterostome δ-catenin clade, but is not robustly grouped with any of the seven subfamilies or any of the three clades identified. The same is true for the single protein members identified in *B. floridae* and *S. purpuratus*, the two other deuterostome lineages included in our study. Finally, all protostomes examined contain a single member of the p120 family and likely are the outgroup of the deuterostome p120 family (Figure 2).

The increase in the number of p120 family members observed in vertebrates and the further increase in fish are consistent with studies suggesting that the ancestral vertebrate underwent two rounds of whole-genome duplication (WGD) [41] and that actinopterygian fish underwent additional rounds of WGD [42], [43]. For example, for every single non-fish vertebrate subfamily member, two or three fish subfamily members are typically identified. However, the increase in number of members is unlikely to have been solely due to the WGDs and additional gene duplications likely contributed to the generation of the current diversity of protein members of the p120 family observed today.

### Kaiso protein family

**Figure 3:**
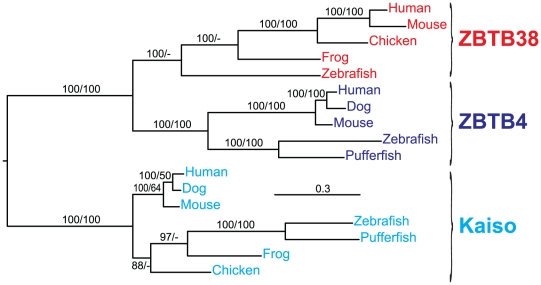
The evolutionary history of the Kaiso family of proteins. The majority-rule consensus tree from a Bayesian inference analysis on an alignment constructed using entire protein sequences is shown. Numbers near internodes indicate posterior probability (for Bayesian inference analyses)/bootstrap (for maximum likelihood analyses) clade support values. Clade support values <50% are not shown.

A total of 17 protein sequences were retrieved from the 14 species examined (Table 2). No Kaiso protein family members were identified in protostomes and in non-vertebrate chordates. All vertebrates contain two or three of the Kaiso family proteins. All vertebrates contain Kaiso, but several are missing either ZBTB4 or ZBTB38 (Table 2). Phylogenetic analysis of all protein family members identified three major clades that correspond to the three proteins (Figure 3) [27].

**Table 2:** Distribution of ZBTB protein family members across animal phyla.

Species	Phylum	Kaiso	ZBTB38	ZBTB4
Human	Vertebrata	✓	✓	✓
Mouse	Vertebrata	✓	✓	✓
Dog	Vertebrata	✓	X	✓
Chicken	Vertebrata	✓	✓	X
Frog (*Xenopus laevis*)	Vertebrata	✓	✓	X
Pufferfish	Vertebrata	✓	X	✓
Zebrafish	Vertebrata	✓	✓	✓
Tunicate	Urochordata	X	X*	X
Amphioxus	Cephalochordata	X	X*	X
Echinoderm	Echinodermata	X	X*	X
Fly	Arthropoda	X	X*	X
Worm	Nematoda	X	X*	X
Polychaete (*Capitella*)	Annelida	X	X*	X
Clam (*Lottia*)	Mollusca	X	X*	X

X*: these proteomes contain many hits with e-values ∼10^−30^.

### Phylogenetic relationships of p120 family to other functionally relevant proteins

**Figure 4:**
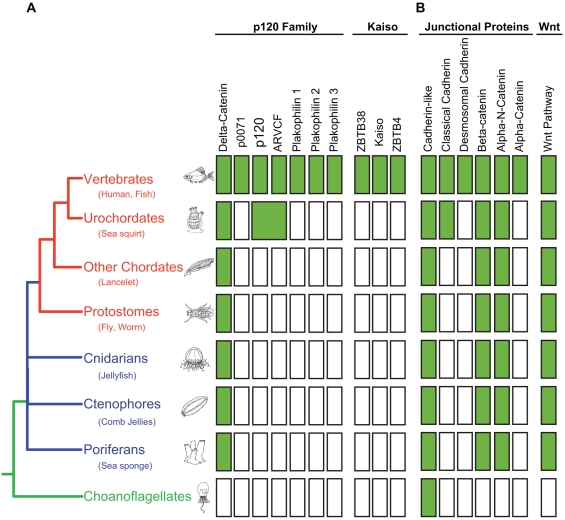
Presence/Absence of families involved in cell-cell adhesion and signalling. Phylogenetic distribution of p120-family proteins, kaiso-family proteins, junctional proteins, and proteins collectively required for a functional wnt pathway. A filled box indicates the presence of an orthologue from the corresponding protein family. Color coding for the phylogenetic tree is as follows: pre-metazoan in green, metazoan in blue, bilateral metazoans in red.

Figure 4 compares our data with respect to phylogenetic histories of the p120 and Kaiso families (Figure 4A) with published [3], [4], [5], [27], [44] and unpublished (i.e., α-catenin family, E. Gaucher, personal communication) histories of other functionally related proteins (Figure 4B). Of particular interest is the existence of a single member of each of the catenin families (i.e.. δ-catenin, α-N-catenin, and β-catenin) conserved throughout pre-vertebrate metazoan evolution, a pattern that coincides with the phylogenetic origins of Wnt family proteins. The plakophillins, on the other hand, along with Kaiso and Desmosomal cadherins, are vertebrate innovations. Of note, the δ-catenin-like and p120-like ancestors of the present day p120 family arise just prior to vertebrates, as does the first common ancestor of the classical cadherins (i.e.. vertebrate Type I and Type II cadherins).

## Discussion

Conservation of a δ-catenin-like gene over the course of metazoan evolution suggests an ancient and evolutionarily important role, but the effects of deleting the only δ-catenin-like gene present in worms and flies is not as dire as one might expect. δ -catenin knockdown in xenopus is, in fact, embryonic lethal [45], but the effects of δ-catenin KO in mice appear to be largely cognitive [46], [47], [48], [49]. Although fly p120/δ-catenin associates and colocalizes with fly E-cadherin [16], the evidence overall suggests that its role is not directly comparable to that of vertebrate p120. A strong possibility is that fly p120/δ-catenin has an ancient function that is nonessential for life but nonetheless confers a strong evolutionary advantage. For example, the significant cognitive abnormalities exhibited by δ-catenin KO mice [46], [49] may not be immediately apparent in captivity but could markedly affect their ability to compete and survive in the wild. Indeed, δ-catenin is one of several genes deleted in human Cri-du-chat patients and may contribute to the mental retardation associated with the disorder [46], [47].

The vertebrate p120 family consists of seven members. Four “core” members (i.e., δ-catenin, p120-catenin, ARVCF, and p0071) function in adherens junctions, and three less well conserved members function in desmosomes (Plakophillins 1, 2, and 3) (Figure 1). The phylogenetic analyses presented here show that they evolved through rounds of gene duplication and functional diversification from an ancient “δ-catenin-like” gene that is conserved throughout metazoan evolution. The ancestral δ-catenin was probably similar in function to the gene member currently present in invertebrates, echinoderms and cephalochordates (Figures 1 and 2; Table 1). The first gene duplication took place in the urochordate-vertebrate ancestor, giving rise to “δ-catenin-like” and “p120-catenin-like” progenitors. Additional gene duplication(s), most likely a consequence of the two rounds of whole genome duplication at the origin of vertebrates, gave rise to (1) a δ-catenin clade consisting of vertebrate δ-catenin and p0071, and (2) a p120 clade consisting of vertebrate p120 and ARVCF. The plakophillins represent a vertebrate specific offshoot of the δ-catenin-like progenitor.

The phylogeny of the p120 family is relatively straight forward, but exactly how or why p120 has evolved to become the predominant family member in vertebrates is harder to explain. One possibility is that p120 has evolved uniquely advantageous features important for cadherin function. Indeed, comparison of current structural and functional characteristics of the various family members reveals several potentially critical p120 adaptations. First, p120 is the only core family member in vertebrates that lacks a C-terminal PDZ ligand domain. This domain mediates protein-protein interactions with a number of important PDZ domain containing proteins (e.g. PSD-950, erbin, densin-180). The PDZ ligand domain itself is an ancient feature of the p120 lineage as it is present, for example, in the sole family member of various protostomes such as *C. elegans*. The p120-like progenitor of p120 and ARVCF, on the other hand, has a C-terminal sequence that differs at one residue from known consensus motif sequences (i.e., NSWV). Notably, the p120 progenitor is equally similar to p120 and ARVCF by most criteria, but a bona fide C-terminal PDZ ligand would imply that the progenitor was functionally more similar to ARVCF than p120.

Regardless, p120 is clearly the only core member of the vertebrate p120 family that lacks the C-terminal PDZ ligand domain and conceivably, certain physical and functional evolutionary constraints imposed by preexisting PDZ binding partners of ARVCF, δ-catenin and p0071. Indeed, spine (and synapse) density in mouse hippocampal neurons is significantly increased by δ-catenin ablation, but the effect is not cadherin-dependent. Instead, it clearly depends on a PDZ-ligand mediated interaction with one or more PDZ domain-containing proteins [49]. In contrast, p120 ablation in the same tissue has the opposite effect on spine density and works through a very different mechanism associated with modulation of Rho GTPases [13]. These data highlight the functional importance of the C-terminal PDZ ligand, and illustrate how it can contribute to the markedly different roles for δ-catenin and p120 in hippocampal neurons, as well as other tissue types. Overall, these observations strongly support the notion that the absence of a PDZ ligand domain may have endowed p120 (and p120 bound cadherin complexes) with significant flexibility to evolve novel physical and functional interactions that are independent of PDZ-mediated roles.

Second, a potentially critical adaptation is the evolution of alternative splicing in the amino-terminal regulatory domain of p120 and ARVCF [50], [51], [52], but apparently not in δ-catenin or p0071. The ability to use alternative start sites allows p120 (and ARVCF) to separately express isoform 1 and/or isoform 3, forms of p120 that likely have significantly different roles. Specifically, isoform 1, but not isoform 3, contains the N-terminal coiled coil (CC) domain, a ∼40 amino acid N-terminal domain that is presumed to be important because it is almost perfectly conserved in all core family members. p120 isoform 1 is expressed predominantly in mesenchymal (e.g., fibroblasts) and certain other non-epithelial cell types (e.g., neurons), whereas the shorter isoform 3 is preferred in epithelial and other relatively sessile cell types. Importantly, p120 isoform switching (e.g., from isoform 3 to isoform 1) is dynamic and typically coordinated with classic cadherin switching (e.g., E-cadherin to N-cadherin) that occurs during epithelial to mesenchymal transformation (EMT) [53], [54]. The ability to directly modulate and/or participate in EMT is likely to be significant, as this process is critically important during development, wound healing and cancer.

Notably, p120 is the only family member possessing both of these innovations (i.e., absence of a PDZ ligand domain and presence of alternative start sites). ARVCF undergoes N-terminal alternative splicing, but contains a C-terminal PDZ ligand motif. Whether one or both of these factors substantially influenced the adoption of p120 by classical cadherins is largely speculation. Nonetheless, if adaptive advantage did in fact play a role, the most likely determinant of such an event is p120 itself, and both factors offer plausible advantages relevant to flexibility and/or function. δ-catenin, on the other hand, is likely constrained by PDZ-mediated interactions and the inability to generate an isoform that lacks the coiled-coil domain.

Interaction with Kaiso provides a third p120 adaptation that is absent from other family members. In support of a previous study by Fillion et al [27], we find that Kaiso is vertebrate-specific, and thus coincides with both the origin of vertebrate p120 and the vertebrate specific expansion of the classical cadherins. Kaiso belongs to a unique family of transcription factors that can associate selectively, and with high affinity, to methylated CpG DNA via zinc finger domains [29]. Kaiso is actually bimodal in that it also binds with lower affinity to a conventional DNA motif [55]. A recent report shows that Kaiso can shut down the transcription of key tumor suppressors (e.g., pRb, p16, Hic1) by interaction with inappropriately methylated CpG islands. Thus, Kaiso may link p120 to epigenetic transcriptional regulation via CpG island methylation, a cancer-relevant and largely vertebrate-specific mechanism associated with the use of hypomethylated CpG islands as sites of active transcription [56]. Interestingly, Kaiso and TCF are reported to associate physically via their DNA binding domains, thereby preventing one another from interacting with chromatin [33]. These data raise the possibility that p120's interaction with Kaiso modulates canonical Wnt signaling through TCF4. While it is unlikely that p120 and Kaiso are essential for Wnt signaling, their influence might be important in the context of complex developmental and regulatory vertebrate environments. Given that Kaiso is absent from non-vertebrate metazoans, the evolution of interactions with both p120 and TCF may represent a vertebrate-specific adaptation connecting cadherin complexes in general, and p120 in particular, to canonical Wnt signaling pathways. What exactly this means for vertebrate Wnt signaling and/or related functions has yet to be determined, but in contrast to β-catenin and TCF, lessons in vertebrate p120 or Kaiso functions are unlikely to be guided by genetic studies in non-vertebrate model systems.

As mentioned, the increase in the number of p120 family members observed in vertebrates is consistent with studies suggesting that the ancestral vertebrate underwent two rounds of whole-genome duplication [41]. Evidently, these were instrumental in the evolution of at least two broad categories of classical cadherin complexes. The ancestral invertebrate forms of α-catenin and p120 were duplicated and have emerged in vertebrates as α-N-catenin and δ-catenin, both of which are found primarily in neural tissues [57], [58], [59], [60]. Their duplicated counterparts, on the other hand, evolved to become α-catenin and p120, respectively, and are expressed in all solid tissues, including the nervous system. In parallel, the classical cadherins evolved from a single vertebrate ancestor by gene duplications that led to the evolution of at least four classical cadherins, most likely the ancestors of present day N-, R-, E- and P-cadherins [4], [5]. These cadherin paralogues appear to represent early neural (N- and R-cadherins) and non-neural/epithelial (E- and P-cadherins) lineages that subsequently evolved at different rates [4]. Thus, in vertebrates, the ancestral “invertebrate counterparts” of p120 and α-catenin (i.e., δ-catenin and α-N-catenin) appear to be primarily confined to the nervous system, while p120 and α-catenin are found in essentially all solid tissues, nervous system included. The very different features of δ-catenin and p120, as discussed above, may account for the relatively restricted tissue specific expression of δ-catenin, and the subsequent emergence of p120 as the most widely expressed member of the p120 family in vertebrates. The apparently analogous co-distribution of the α-catenin isoforms (E. Gaucher, personal communication) is probably related to these events, although coordinated alterations in gene regulatory elements could contribute to such events in any of the scenarios described above.

In any event, these observations suggest that in most vertebrate tissues, the main functional unit defined by the present-day classical cadherin complex came together for the first time as a result of whole genome duplications that caused the ancestral catenins (δ-catenin and α-N-catenin) to partition with the neuronal lineage, (presumably in association with N-cadherin), and their vertebrate-specific derivatives (p120 and α-catenin) to form a second lineage (presumably in association with a common ancestor of non-neural cadherins - perhaps E- and/or P-cadherins), which was then favored as the raw material for diversification of most other tissues. The former was likely constrained by the need to conserve complex neuronal functions whereas the rapid evolution of the latter is consistent with a cadherin complex that is more flexible with respect to expansion of novel interactions. The extraordinary success of this ultimate classical cadherin complex is evidenced by the repeated duplication and diversification of the classical cadherins to at least 26 members, most of which use the same basic set of p120-, α- and β-catenin building blocks. This core design has thus been preserved and reutilized by classical cadherins for approximately half a billion years, while simultaneously serving as a key driver of vertebrate cell- and tissue-diversification.

Interestingly, a similar paradigm appears to extend to the desmosomal cadherins and their interaction with the more distant members of the p120 family, the plakophillins. Figure 2 shows that the plakophillins are of vertebrate origin and share a common ancestor with the vertebrate δ-catenin clade. Their appearance coincides with that of several other important components of desmosomes, which also originate in vertebrates. Plakoglobin, for example, evolved around the same time via gene duplication of β-catenin, and functions in both adherens junctions and desmosomes. Interestingly, the desmosomal cadherins later diverge from other cadherins and the family appears to expand within mammals [5], permitting evolution of the desmosome. Our analyses also show that the plakophilins are the fastest evolving members of the p120-family (Figure 2). Importantly, like p120 and β-catenin, the plakophillins also have roles in the nucleus [51], [61], [62], [63], [64], suggesting other potentially significant functions that have yet to be defined. Overall, the fastest evolving clade of the p120 family and the desmosomal cadherins appear to be recycling the evolutionary game plan of the classical cadherins.

## Materials and Methods

### Data matrix construction

The complete proteome sequence files of 7 vertebrates (Homo sapiens, Canis familiaris, Mus musculus, Gallus gallus, Xenopus tropicalis, Danio rerio, and Takifugu rubripes), 1 urochordate (Ciona intestinalis), 1 cephalochordate (Branchiostoma floridae), 1 echinoderm (Strongylocentrotus purpuratus) and 4 protostomes (Drosophila melanogaster, Caenorhabditis elegans, Helobdella robusta, and Lottia gigantea) were retrieved from the Ensembl FTP Server (http://uswest.ensembl.org/info/data/ftp/index.html) and JGI Genome Portal (http://genome.jgi-psf.org/) websites. All proteome sequence files were processed so that only the longest protein sequence product of a given gene was retained using a custom Perl script. Members of the δ-catenin protein family were identified using the blastp similarity search algorithm, version 2.2.16 [65]. This was done by blasting the human p120 protein (genbank accession number: NP_001078927.1) against each proteome and retrieving all protein sequences showing significant similarity. Similar results were obtained using other members of the δ-catenin protein family from Homo sapiens or from other species.

### Phylogenetic analyses

Phylogenetic analyses were performed using the optimality criteria of Bayesian inference (BI) and Maximum Likelihood (ML). According to the BI optimality criterion, the tree that best explains our protein alignment is considered the best estimate of the true phylogeny of our proteins [66]. According to the ML criterion, the tree that makes our protein alignment the most probable evolutionary outcome given a specific model of protein evolution is considered the best estimate of the true phylogeny of our proteins [66]. BI and ML analyses were performed on two data matrices: the first data matrix was generated by the alignment of whole proteins, whereas the second data matrix was generated by concatenating the individual alignments of each of the nine Arm domains. All alignments were constructed using dialign, version 2.2 [67]. dialign is a local alignment algorithm that does not attempt to align proteins from start to finish. Instead, it only aligns the conserved protein regions between proteins and identifies all remaining (poorly conserved) regions as unaligned. This feature is particularly useful for aligning proteins like the p120 family where conserved domains are flanked by poorly conserved regions of varying length. Importantly,dialign displays all aligned residues in capital letters, and all unaligned residues in lowercase letters. In all cases, all unaligned amino acids were converted to “X”, the IUPAC symbol for unspecified amino acids, and were effectively filtered out from downstream phylogenetic analyses. Sequences belonging to each Arm domain were manually identified through careful comparison with the human p120 protein sequence. Alignments of each of the nine Arm domains were done in exactly the same fashion. BI analyses were conducted using mrbayes, version 3.1.2 [68], [69], [70]. BI phylogenetic trees were constructed using a mix of empirical amino acid substitution matrices, allowing for rate of heterogeneity among sites by assuming that a certain proportion of sites were invariable and that the rates of the rest are determined according to the shape parameter alpha of the gamma distribution. Two independent analyses were run in parallel. Each analysis contained 4 chains (1 cold and 3 incrementally heated) and trees were sampled every 1,000 generations. The analyses were run for 2,000,000 generations by which time the average deviation of split frequencies was below 0.01. The trees and parameters sampled from the first 10% of generations from each of the two analyses were discarded as the burn-in. Clade support in BI analyses was assessed using posterior probabilities. ML analyses were conducted using raxml, versions 7.2.5 and 7.2.6 [71]. ML phylogenetic trees were constructed using the WAG amino acid matrix [72], allowing for rate of heterogeneity among sites by assuming that the rates of the rest are determined according to the shape parameter alpha of the gamma distribution. Clade support in maximum likelihood analyses was assessed using non-parametric bootstrap re-sampling (100 replicates).
